# Fractal features of soil grain-size distribution in a typical *Tamarix* cones in the Taklimakan Desert, China

**DOI:** 10.1038/s41598-022-20755-x

**Published:** 2022-09-30

**Authors:** Zhengwu Dong, Donglei Mao, Mao Ye, Shengyu Li, Xiaodong Ma, Suiyunhao Liu

**Affiliations:** 1 College of Life Science, Xinjiang Normal University, Ürümqi, 830046 People’s Republic of China; 2 College of Geographic Science and Tourism, Xinjiang Normal University, Ürümqi, 830046 People’s Republic of China; 3 Xinjiang Institute of Ecology and Geography, Chinese Academy of Sciences, Ürümqi, 830011 People’s Republic of China; 4 Xinjiang Key Laboratory of Special Species Conservation and Regulatory Biology, Ürümqi, 830054 People’s Republic of China

**Keywords:** Ecology, Environmental sciences, Solid Earth sciences

## Abstract

*Tamarix* cones play key roles in preventing sand erosion and maintaining regional ecosystem stability. This study aimed to explore the characteristics of soil grain size distribution (GSD) in *Tamarix* cones across the Taklimakan Desert, verify the relationships between soil grain composition and the fractal dimension, and analyze the relationships between soil GSD and environmental factors. Soils of the *Tamarix* cones from 0 to 500 cm soil depth were sampled every 20 cm at four sites (Qiemo, Qira, Aral, and Tazhong) along the periphery to the hinterland of the Taklimakan Desert. A total of 300 soil samples were collected to measure soil grain sizes and soil properties. Soil grain size composition was dominated by silt and very fine sand, and the fraction of fine particles decreased and that of the coarse particles increased with soil depth, except for at Tazhong. This suggested that suspension-size particles are the main component of the soil GSD and decrease with the increasing depth in the profiles at the *Tamarix* cones. The soils were poorly and moderately poorly sorted. Kurtosis generally showed a mesokurtic peak, and the GSD was negatively skewed towards the coarser particles. The fractal dimensions of GSD decreased in the following order Qiemo (2.30) > Qira (2.07) > Aral (1.99) > Tazhong (1.96) because of the increase of coarse particles. The fractal dimension had a strong positive correlation with the clay and silt fractions, and a strong negative correlation with the sand fraction, indicating that the fractal dimension can reflect the characteristics of the soil GSD. The strong relationships between the fractal dimensions and selected soil properties indicate that the fractal dimension can not only quantify changes in soil properties, but also reflect the degree of desertification and degradation in the desert region. Considering the strong wind activities and different deposition sources in the Taklimakan Desert, this study provides a deep insight into the soil formation processes of *Tamarix* cones within extreme arid desert ecosystems.

## Introduction

The soil grain size distribution (GSD) is a crucial soil physical property that can not only affect the composition and structure of soil, but also have a great influence on soil fertility, erosion, and the retention and movement of soil water^1–3^. Furthermore, soil GSD is considered a good indicator for the evaluation of soil structures and functions^4,5^. A quantitative description of GSD is therefore vital for research into soil formation mechanisms.

Several methods have been developed to characterize soil GSD^6–9^. Textural analysis is the most widely used approach; however, the size definitions of three primary grain compositions (clay, silt, and sand) are rather arbitrary^10^. To better explain the relationship between soil GSD and other factors, the fractal theory and relevant parameters (average grain size, sorting coefficient, kurtosis, and skewness) have been applied to soil systems. Previous studies have shown that the average grain size and skewness can reflect the range of soil GSD, while the sorting coefficient and kurtosis mainly characterize the dispersive degree of soil GSD^11,12^. Moreover, fractal theory has frequently been applied to soil science because the soil exhibits fractal features. Many studies have used the fractal dimension to characterize soil GSD, structure, and dynamics, because it allows for a better understanding of soil physical processes^13–15^. Therefore, a clear understanding of soil GSD as well as its parameters is necessary to comprehend soil formation processes and to evaluate the role of aeolian deposition in desert ecosystems.

Previous studies have indicated that the fractal dimension is very sensitive to soil particle composition. It increases with the increase of clay and silt content but decreases with the increase of sand content^16,17^. In addition, the fractal dimension has been shown be significantly positively correlated with soil fine particles (< 0.05 mm) and organic matter contents in desert areas^18^. The fractal dimension of soil GSD can characterize soil texture homogeneity, nutrient contents, and permeability, and also reflect changes to the soil properties following environmental changes and human activities^19,20^. In particular, the soil fractal dimension can be applied to monitor soil erosion and degradation induced by human activities and climate changes. It can also be used to evaluate soil evolution processes and the degree of soil desertification in desert environments^7,14^. Therefore, investigating the characteristics and factors affecting soil GSD in desert regions is critical for understanding of soil formation processes and environmental changes in desert ecosystems.

*Tamarix taklamakanensis* is the dominant shrub species in the Taklimakan Desert, and it grows on sand dunes and forms the so-called *Tamarix* cones (*Tamarix*-cone microtopography). *Tamarix* cones have heights ranging from 3 to 15 m and lengths from 5 to 50 m (long axis)^21^. They are mainly distributed in the lower reaches of the Tarim River, the transitional zones between desert and ambient oases and the hinterland of the Taklimakan Desert^22^. However, the degeneration and extinction of *Tamarix* cones have appeared in desert ecosystems due to the human activities and climate changes, which may directly affect the stability of the local desert ecosystem^23^. Previous studies of *Tamarix* cones have mainly focused on the formation mechanism, morphological structure, environmental indicators, and the nutrient contents and salinity of the soil surface^24–28^. The study of Dong et al.^28^ indicated that The soil particle size composition of *Tamarix* cones is dominated by fine particles (clay and very fine sand), with a content of more than 80%, and fine suspension particles (i.e., < 50 μm) generally increased with increasing soil depth, whereas saltation particles (50–500 μm) decreased with depth in the Gurbantunggut Desert. Moreover, the fractal dimension and average particle size have been found to be significantly positively correlated with clay and silt fraction, and significantly negatively correlated with very fine sand and fine sand fraction, indicating that fractal dimension and average particle size can reflect soil particle size composition of *Tamarix* cones. Some studies have also shown that soil grain size composition in the upper soil of the *Tamarix* nabkha is dominated by the very fine sand and silt in the oasis–desert ecotone^29^. Furthermore, the decrease of vegetation coverage and the aggravation of wind–sand erosion directly result in the average grain size increased in the surface soil of *Tamarix* nabkha, with clay and silt loss and coarse sand appearing in the upper soil^30,31^. In addition, soil GSD has been found to be closely related to soil properties (e.g., soil water content, organic matter, total phosphorus, and electrical conductivity), environmental factors (e.g., climate and topography) and anthropogenic activities in the formation of coppice dunes^32,33^. However, this is a crucial research gap, considering the importance of fractal dimension in soil grain size composition patterns and the influence of soil properties in soil GSD and soil forming processes in *Tamarix* cones.

In this study, we analyzed the soil GSD and its parameters (average grain size, sorting coefficient, kurtosis, and skewness) in *Tamarix* cones and explored the relationships between soil GSD and environmental factors. We hypothesized that suspension-size particles are the main component of the soil GSD and decrease with the increasing depth in the profiles at the *Tamarix* cones, and that the fractal dimension can reflect the characteristics of the soil GSD. In addition, aeolian depositions are the main driving factors for the formation of *Tamarix* cones in the Taklimakan Desert^29^. In addition to the influence of wind erosion and sand sources, we hypothesized that soil GSD and fractal dimension are closely related to soil nutrient, soil water content and soil salinity in the *Tamarix* cones. The objectives of the study were: (1) to characterize the detailed soil grain-size distribution and the parameters of *Tamarix* cones for four study sites, (2) to investigate the relationship between soil particle size composition, selected soil properties, and fractal dimension, and (3) to analyze the influence of environmental factors on soil particle size composition. This study will help to clarify the soil formation processes of *Tamarix* cones. Moreover, the findings of our study will contribute to improving the protection and management of coppice dunes in desert areas.

## Materials and methods

### Study area

The study area is located at the central and peripheral areas of the Taklimakan Desert in NW China (37°–41°N, 77°–90°E), which comprises ~ 85% shifting sand dunes. This region is an important dust source area with annual average dusty days reaching up to 100^29,34^. The Taklimakan Desert is extremely arid, with the mean annual precipitation ranging from 24.0 to 65.3 mm from east to west, and annual average evaporation reaching up to 3000 mm^29^. The initial desert landscape occurred well before the Pleistocene, and desertification was enhanced under cold, dusty Pleistocene glacial conditions^35^. The soil is mainly composed of brown desert soil (normally dry soil) and aeolian sandy soil (new sandy soil), the sand supply for the dunes is mainly from local alluvial or lacustrine deposits^36^. Because of moisture limitation, the vegetation is scarce in this region, and the predominant species are mainly shrubs, including *Tamarix taklamakanensis*, *Populus euphratica*, *Karelinia caspia*, and *Alhagi sparsifolia.*

**Figure 1:**
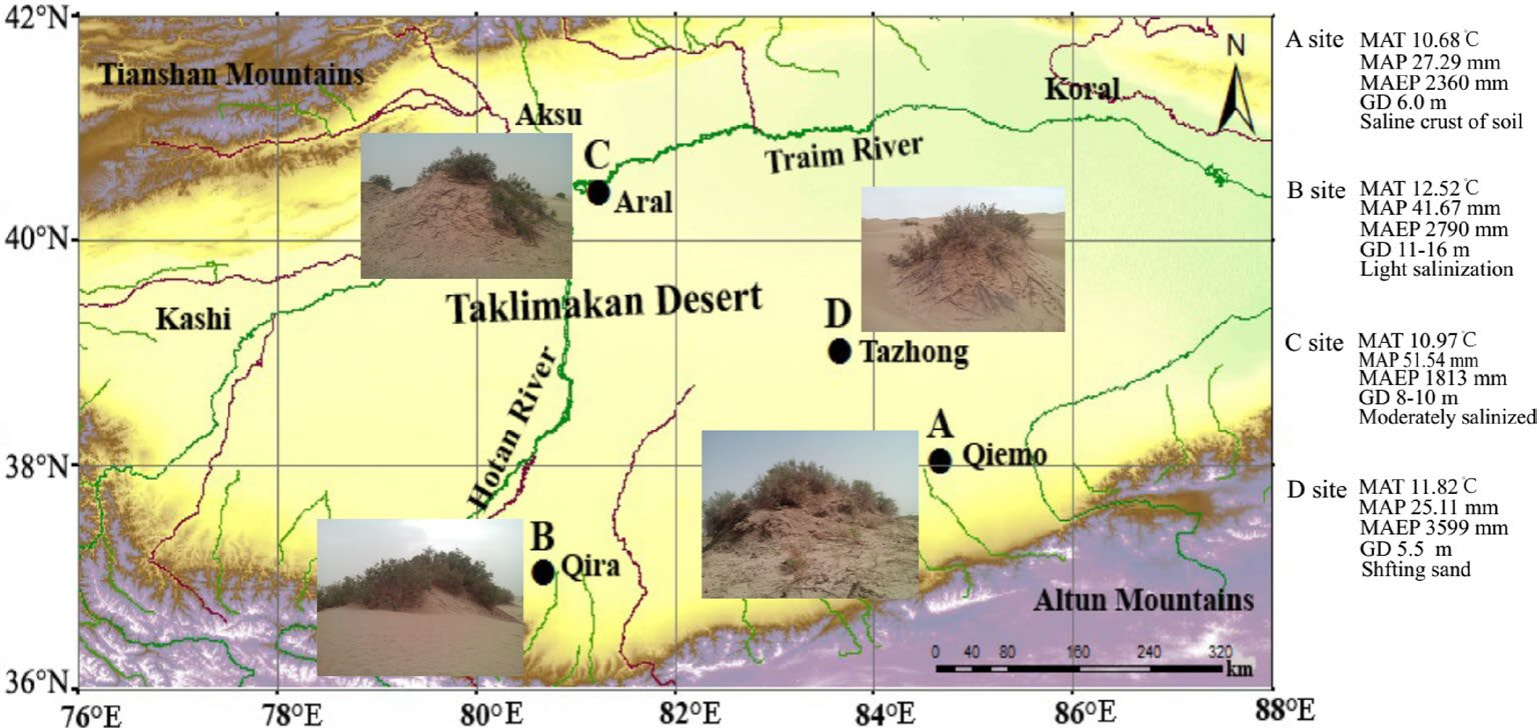
Location map of sampling plots in the Taklimakan Desert (where A, B, C, and D represent different habitats of *Tamarix* cones. A: Saline desert–alluvial plain of Qiemo; B: oasis–desert ecotone of Qira; C: oasis–desert ecotone of Aral; and D: Sandy desert Habitat of Tazhong. MAT = mean annual temperature; MAP = mean annual precipitation; MAEP = mean annual evaporation; GD = Groundwater depth. The map was created using ArcGIS10.7 software (https://www.onlinedown.net/soft/1162654.htm) and Adobe Illustrator (AI) CS5 software (https://www.onlinedown.net/soft/578049.htm).

Four typical *Tamarix* cone sites were selected in the Taklimakan Desert, based on soil and vegetation cover conditions. Sampling plot A was located near Qiemo in the southeast fringe of Taklimakan Desert along the desert highway. The vegetation coverage was about 50% in the *Tamarix* cones, and the study area was in a transition zone between alluvial plain and desert, where the saline soil and strong evaporation in summer contributed to the formation of salt crust at the soil surface. Sampling plot B was situated in the southern fringe of the Taklimakan Desert, about 10 km from Qira county, and represents an oasis–desert ecotone with a *Tamarix* cone plant cover of 40%, and the soil surface was dominated by shifting sand with light salinization. The dominant wind was from the west, with an occurrence frequency of 62.3–76.23%, and westerly dust-raising winds (> 6 m/s) accounted for 94.6% of the total. The mean annual numbers of dust storm and dust days were 21.2 and 142.4, respectively^28^. Sampling plot C was located near the city of Aral in the northern fringe of the Taklimakan Desert. The plant cover of *Tamarix* cones was 30% and the soil surface was moderately salinized and covered by a thin crust. Sampling plot D was located near the Tazhong Desert Station. The plant cover was about 15% on the *Tamarix* cones. At the surface of the *Tamarix* cones, there was scattered shifting sand. The mean wind speed is 2.5 m/s, and the highest instantaneous speed reaches 24.0 m/s. The mean annual sand and dust weather reaches 260 days^30^. The main soil texture of all four sampling plots was aeolian sandy. These sampling plots were selected to cover the typical range of *Tamarix* cone habitats within the Taklimakan Desert (Fig. 1). For the ease of reference to the sampling plots in the study area, the four sampling plots of *Tamarix* cones labeled A, B, C, and D were designated the location names, Qiemo, Qira, Aral, and Tazhong, respectively.

### Sample collection and analysis

At each sampling site, three mature individual *Tamarix* cones of similar coverage and size were selected for soil sampling. The adjacent sampling cones were about 500 m apart. At all sampling sites, average height and basal diameters for the *Tamarix* cones were 3.23 ± 0.30 m and 10.67 ± 0.39 m, respectively. Sampling was conducted in July 2018. The soil samples were obtained from three *Tamarix* cones at each site using a hand auger. A total of 12 soil cores were collected from the four habitats. At each sampling site, 25 soil samples were collected at 20 cm intervals down to 500 cm depth. The soil samples were sealed in polythene bags and taken to the laboratory. All samples were air-dried, one part sieved through a 2-mm mesh by hand to remove roots and other detritus for GSD analysis, and the other part was passed through a 0.15-mm mesh for chemical analysis.

Soil GSD was analyzed by the laser detection technique on a Malvern Mastersizer 2000 (Malvern Instruments, Malvern, England). The measurement error was less than 2%. Soil samples were pretreated using 30% H_2_O_2_ and 10% HCl to remove organic matter and carbonate, respectively. Subsequently, the aggregates were dispersed using 10 ml of 0.05 mol/L sodium hexametaphosphate (NaHMP) and ultrasonics lasting for 30 s. Soil grain diameters were in the range of 0.02 to 2000 μm^5^. According to the US classification standards, the soil GSD was described in terms of the percentages of clay (< 0.002 mm), silt (0.002–0.05 mm), very fine sand (0.05–0.1 mm), fine sand (0.1–0.25 mm), medium sand (0.25–0.5 mm), and coarse sand (0.5–1.0 mm).

Soil pH was measured using a digital pH meter (pHS-2C, Precision and Scientific Corp. Shanghai, China), and soil EC was measured using a conductivity meter (DDS-307A, Precision and Scientific Corp. Shanghai, China) in 1:5 soil:water solution. The SOM was analyzed using the Walkley–Black modified acid–dichromate FeSO_4_ titration method. The STN was measured after micro-Kjeldahl digestion using a flow injection auto-analyzer. The STP was determined calorimetrically by the ammonium molybdate method. The SWC was measured by the oven drying method. All the methods were referenced from Bao^37^.

### Calculation of grain size parameters

The average grain size (*M*_*G*_*/*μm), sorting coefficient (*σ*_*G*_), skewness (*SK*_*G*_), and kurtosis (*K*_*G*_) were calculated as follows Folk and Ward^38^:

1 $$M_{G} = \frac{{\phi_{16} + \phi_{50} + \phi_{84} }}{3}$$

2 $$\sigma_{G} = \frac{{\phi_{84} - \phi_{16} }}{4} + \frac{{\phi_{95} - \phi_{5} }}{6.6}$$

3 $$SK_{G} = \frac{1}{2}\left( {\frac{{\phi_{84} + \phi_{16} - 2\phi_{50} }}{{\phi_{84} - \phi_{16} }} + \frac{{\phi_{95} + \phi_{5} - 2\phi_{50} }}{{\phi_{95} - \phi_{5} }}} \right)$$

4 $$K_{G} = \frac{{\phi_{95} - \phi_{5} }}{{2.44(\phi_{75} - \phi_{25} )}}$$

where $\phi_{5}$, $\phi_{16}$, $\phi_{25}$, $\phi_{50}$, $\phi_{75}$, $\phi_{84}$ and $\phi_{95}$ are the grain sizes (in phi) of the different frequencies. The grain sizes were categorized into three fractions: sand (> 63 μm), silt (63–2 μm), and clay (< 2 μm)^39,40^.

### Estimation of the fractal dimension

The soil grain volume fractal model was used, and the fractal dimension (D) value was calculated by the method of Tyler and Wheatcraft^41^:

5 $$\frac{{V_{{\text{i}}} }}{{V_{T} }} = \left( {\frac{{d_{i} }}{{d_{\max } }}} \right)^{3 - D}$$

where *V*_*i*_ is the total volume of grains with a size less than *d*_*i*_; *V*_*T*_ is the total volume percentage of soil grains; *d*_*i*_ is the mean grain diameter of soil between two adjacent grains sizes *d*_*i*_ and *d*_*i*+*1*_; and *d*_*max*_ is the mean diameter of the largest soil grains. Taking logarithms on both sides of Eq. (5), the D value can be derived from the slope of the logarithmic linear regression equation^20^.

### Data analysis

One-way analyses of variance (ANOVA) was used to analyze the soil GSD and their parameters with soil depth at different sites. Figures were plotted using the software Origin 2017 (Origin Lab Corp., Northampton, USA). The regression analysis and Pearson’s correlation were used to analyze the relationships between the fractal dimensions and soil properties. Distance-based redundancy analysis (db-RDA) was used to explore the influence of selected environmental factors on soil GSD. The ANOVA, db-RDA, Pearson correlation, and regression analysis were performed in R 3.5.2. Meteorological data (precipitation, temperature, and evaporation) were obtained from the Meteorological Information Center of Xinjiang and China meteorological data network (http://data.cma.cn/data/weatherB-k.html).

### Ethics approval and consent to participate

The research using field studies of collected plant material, comply with relevant institutional, national, and international guidelines and legislation. The plant material used in this study was provided by Xinjiang Institute of Ecology and Geography, Chinese Academy of Sciences. *Tamarix* is a common plant extensively cultivated in the world. This study does not contain any research requiring ethical consent or approval. No specific permits are required for sample collection in this study. This article did not contain any studies with wild species at risk of extinction.

## Results

### Characteristics of the soil GSD

**Figure 2:**
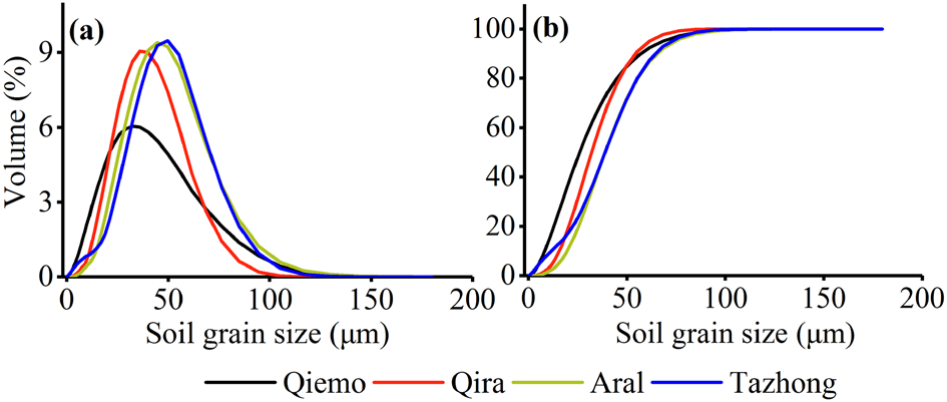
Variation characteristics of the soil grain size distribution at the different sites.

The distribution of soil grain size spanned from 0.19 to 200 μm at the four sites (Fig. 2). The predominant soil grain size was found to be silt and very fine sand. The silt content ranged from 60.60 to 79.14% and that of very fine sand ranged between 19.14 and 37.52%. The contents of clay and fine sand were relatively lower, with the content of clay ranging from 0 to 4.81%, and that of fine sand from 0 to 2.27% (F = 18.57, *p* < 0.01). At Qiemo and Qira, the high peak values of GSD occurred at 32.41 and 36.07 μm, respectively. However, soil GSD showed highly similar unimodal curves at Aral and Tazhong, and the largest peak in the distribution occurred at 44.69 and 49.74 μm, respectively (Fig. 2a). In addition, the silt content was significantly higher than that of other grain sizes at all sites (F = 16.81, *p* < 0.01). The contents of fine components (< 50 μm in diameter) at Qiemo (84.63%) and Qira (84.95%) were significantly higher than those at Aral (71.51%) and Tazhong (71.28%) (F = 10.05, *p* < 0.01) (Fig. 2b). According to the US soil texture size classification standard, the silt contents at Qiemo (79.14%) and Qira (77.52%) were significantly higher than those at Aral (62.04%) and Tazhong (60.60%) (F = 13.95, *p* < 0.01). The contents of very fine sand at Qiemo (19.41%) and Qira (22.43%) were significantly lower than those at Aral (36.68%) and Tazhong (37.52%) (F = 9.17, *p* < 0.01). These results indicate that the difference in the content of fine particle matter (silt and very fine sand) represents the primary difference in the soil texture of the four sites, and the Aral and Tazhong sites had a relatively coarser soil texture than other sites.

When considering different soil depths, all soil layers had unimodal distributions except for 400–500 cm (Fig. S1). Nevertheless, at Tazhong, soil GSD had a bimodal distribution at 400–500 cm depth, with the peaks clustered at 8.97 and 40.15 μm. The peak values of GSD at 0–200 and 200–400 cm soil depth were 47.22 and 52.55 μm, respectively, and the peaks changed little from 0–200 to 200–400 cm at Tazhong. At Qiemo, the peak values (34.24 μm) were consistent among the 0–100, 100–200, and 300–400 cm layers, and the peak values (38.11 μm) were also consistent between the 200–300 and 400–500 cm layers. At Qira, the peaks clustered at 34.24 μm at 0–400 cm; however, the peaks clustered at 38.11 μm at 400–500 cm. At Aral, the peaks appeared in 47.22 μm among the 0–100 and 200–500 cm layers, and clustered at 52.55 μm in the 100–200 cm layer (Fig. S1). These findings indicate that the grain size increased with soil depth.

### Characteristics of soil grain size

**Figure 3:**
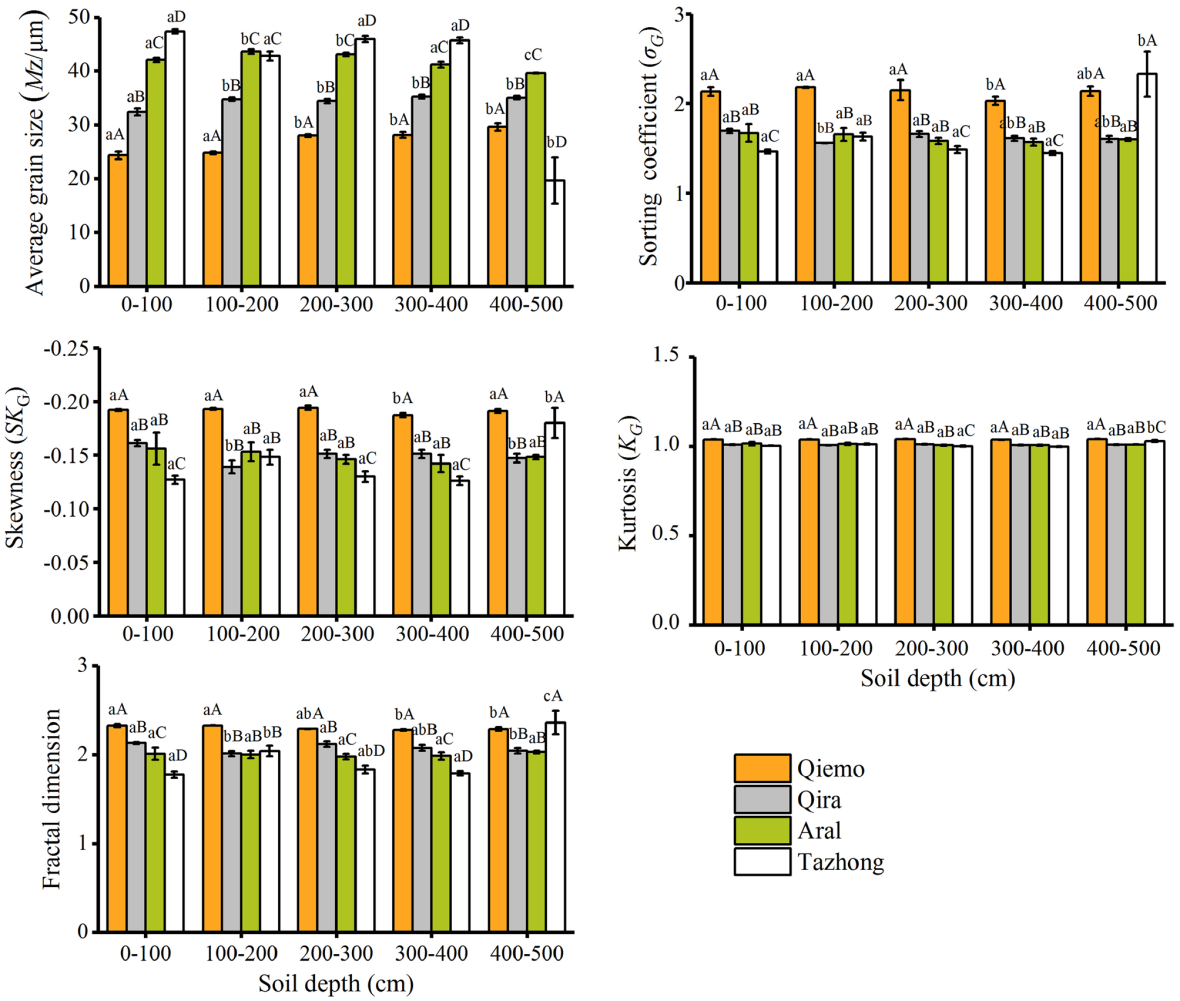
Grain-size parameters of soil at the different depths.

The single fractal dimension of the soil GSD ranged from 1.77 to 2.36 μm in the different habitats (Fig. 3). The fractal dimension decreased in the following order: Qiemo (2.30) > Qira (2.07) > Aral (1.99) > Tazhong (1.96), indicating the fineness of soil texture in the Qiemo was significantly higher than that of the other sites. At Qiemo, the largest fractal dimension were found in the surface soil layer, and subsequently decreased with increasing soil depth. At Qira, the largest fractal dimension were found in the 0–100 cm soil layer, and the fractal dimension decreased initially (0–200 cm) then increased at 200–300 cm, then decreased again at 300–500 cm. At Aral, there was no significant difference in fractal dimension along the soil profiles. However, at Tazhong, the largest fractal dimension were found in the 400–500 cm soil layer; the fractal dimension increased initially (0–200 cm) and then decreased with soil depth (200–400 cm), but increased again at 400–500 cm.

The mean grain size increased with soil depth at Qiemo and Qira, and the mean grain size ranged from 24.33 to 29.62 μm and from 32.37 to 35.23 μm, respectively. At Aral, the mean grain size was 42.10 μm and the smallest mean grain size (39.59 μm) was found at 400–500 cm, with relatively little variation in average grain sizes from 0 to 400 cm soil depth. At Tazhong, the mean grain sizes were considerably changed from the surface soil to the deep soil. The largest mean grain size (47.33 μm) was found in the 0–100 cm layer and the smallest mean grain size (19.64 μm) was found in the 400–500 cm layer.

**Figure 4:**
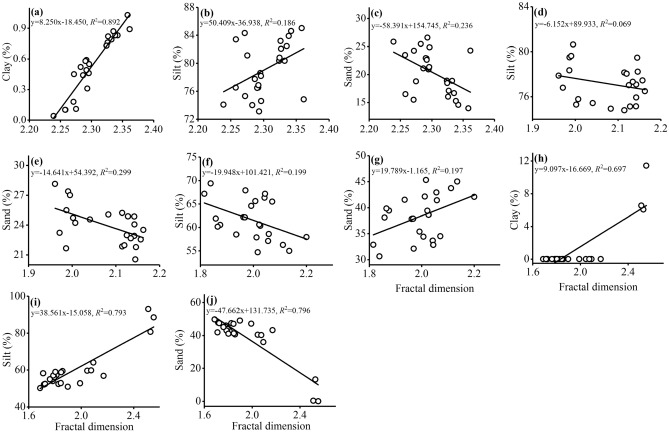
Linear regression relationships between the fractal dimension and the clay, silt, and sand contents at the different sites (Qiemo: **a**–**c**, **a**: r = 0.94, *p* < 0.01, n = 25; **b**: r = 0.43, *p* < 0.05, n = 25; **c**: r = − 0.49, *p* < 0.05, n = 25; Qira: **d**,**e**, **d**: r = − 0.01, p > 0.05, n = 25; **e**: r = − 0.01, *p* > 0.05, n = 25; Aral: **f**,**g**, **f**: r = − 0.45, *p* < 0.05, n = 25; **g**: r = 0.45, *p* < 0.05, n = 25; and Tazhong: **h**–**j**, **h**: r = 0.75, *p* < 0.01, n = 4; **i**: r = 0.79, *p* < 0.01, n = 25; **j**: r = − 0.79, *p* < 0.01, n = 24).

The sorting values were between 1.45 and 2.33 for the four sites of *Tamarix* cones (Fig. 4). The soil was poorly sorted with soil depth at Qiemo. At Qira and Aral, the soil was moderately poorly sorted with soil depth. However, at Tazhong, the soils at 0–400 cm and 400–500 cm depth were moderately poor and poorly sorted, respectively. The GSD exhibited a negative skewness at all sites. Kurtosis values ranged from 0.99 to 1.04 at all sites, which represents a mesokurtic peak.

### Relationship between the fractal dimension and the soil GSD

Linear regression and Pearson correlation analyses were performed to determine the strength of the relationships between the fractal dimension and soil GSD in the different habitats (Table 1, Fig. 4). The fractal dimension of GSD had a strong positive correlation with the clay and silt contents (*p* < 0.05), and a negative correlation with the sand content (*p* < 0.05) at Qiemo and Tazhong (Table 1, Fig. 4a–c,h–j). However, the fractal dimension of GSD had a weak negative correlation with the content of silt and sand at Qira (Table 1, Fig. 4d,e). At Aral, the fractal dimension of GSD had a negative correlation with the silt content (*p* < 0.05), and a positive correlation with the sand content (*p* < 0.05) (Table 1, Fig. 4f,g). In addition, the fractal dimension of GSD had a strong positive correlation with the clay and silt contents, and a strong negative correlation with the sand content at all studied soil depths (Table 1), suggesting that the fractal dimension of GSD increases as soil becomes finer in texture.

**Table 1:** Correlation coefficients between the clay, silt, and sand contents and the fractal dimension.

Grain size	Sites				Soil depth (cm)				
	Qiemo	Qira	Aral	Tazhong	0–100	100–200	200–300	300–400	400–500
Clay	0.944***	–	–	0.745***	0.676**	0.856***	0.749***	0.611**	0.569**
Silt	0.431*	– 0.010	– 0.446*	0.796***	0.813***	0.520*	0.843***	0.841***	0.633**
Sand	– 0.488*	– 0.010	0.446*	– 0.795***	– 0.818***	– 0.536*	– 0.849***	– 0.844***	– 0.652**

*p < 0.05, **p < 0.01, ***p < 0.001.

### Relationships between soil GSD, fractal dimension, and selected soil properties

The fractal dimension had a significant positive correlation with soil organic matter, soil total nitrogen, and soil water content at 0–500 cm soil depth. SWC exhibited a strong positive correlation with clay and silt, and a significant negative correlation with sand at all soil depths (*p* < 0.05) (Table2). soil organic matter, soil total nitrogen, soil litters content, and electrical conductivity had significant positive correlations with clay and silt contents, and significant negative correlations with sand in the 0–400 cm soil layers (*p* < 0.05). Soil pH had a significant negative correlation with clay and silt contents, and a significant positive correlation with sand content in the 0–400 cm soil layers (*p* < 0.05). These results indicate that soil grain distribution was affected by selected soil properties. However, in the 400–500 cm layer, there was no significant correlation between clay, silt, and sand contents and selected soil properties, except for soil total phosphorus and soil water content, indicating that the soil particles in the deeper soil were affected by soil total phosphorus and soil water content.

**Table 2:** Correlations among the fractal dimension, clay, silt, and sand contents and selected soil properties at different soil depths.

Soil depth (cm)	Grain size	SOM	STN	STP	SLC	SWC	pH	EC
0–100	Clay	0.943***	0.819***	0.093	0.473*	0.529*	– 0.780***	0.930***
	Silt	0.713***	0.796***	0.595**	0.156	0.504*	– 0.571**	0.692***
	Sand	– 0.726***	– 0.805***	– 0.589**	– 0.166	– 0.510*	0.582**	– 0.706***
	Fractal dimension	0.817***	0.913***	0.666**	0.227	0.559*	– 0.671**	0.793***
100–200	Clay	0.940***	0.932***	– 0.113	0.986***	0.846***	– 0.875***	0.930***
	Silt	0.622**	0.754***	0.392	0.625**	0.632**	– 0.531*	0.494*
	Sand	– 0.638**	– 0.768***	– 0.381	– 0.643**	– 0.645**	0.547*	0.512*
	Fractal dimension	0.831***	0.858***	0.012	0.850***	0.760***	– 0.737***	0.836***
200–300	Clay	0.871***	0.853***	0.341	0.752***	0.588**	– 0.844***	0.886***
	Silt	0.626**	0.812***	0.788***	0.408	0.681***	– 0.420	0.536*
	Sand	– 0.638**	– 0.821***	– 0.786***	– 0.420	– 0.685***	0.434	– 0.550*
	Fractal dimension	0.759***	0.879***	0.747***	0.589**	0.797***	– 0.537*	0.743***
300–400	Clay	0.786***	0.767***	0.093	0.927***	0.444*	– 0.586**	0.757***
	Silt	0.713***	0.813***	0.616**	0.472*	0.935***	– 0.827***	0.674**
	Sand	– 0.720***	– 0.819***	– 0.613**	– 0.482*	– 0.935***	0.830***	– 0.680**
	Fractal dimension	0.802***	0.849***	0.552*	0.629**	0.836***	– 0.942***	0.817***
400–500	Clay	0.061	0.066	– 0.665**	– 0.122	0.877***	0.385	– 0.121
	Silt	0.412	0.360	– 0.222	0.058	0.797***	0.017	0.056
	Sand	– 0.339	– 0.378	0.357	– 0.012	– 0.866***	– 0.120	– 0.010
	Fractal dimension	0.559*	0.592**	– 0.349	0.305	0.516*	– 0.163	0.430

*SOM* soil organic matter, *STN* soil total nitrogen, *STP* soil total phosphorus, *SLC* soil litter content, *SWC* soil water content, *EC* electrical conductivity.

*p < 0.05, **p < 0.01, ***p < 0.001.

Soil organic matter, soil total nitrogen, and electrical conductivity had a significant correlation with clay, silt, and sand contents at all sites (*p* < 0.05) (Table S1). This shows that soil organic matter, soil total nitrogen, and electrical conductivity were the main factors affecting the soil GSD. Furthermore, we found that soil pH and soil litters content had a strong correlation with clay, silt, and sand contents at Qiemo. soil water content was significantly correlated with clay, silt and sand contents at Qira and Tazhong. The fractal dimension had a strong correlation with soil organic matter, soil total nitrogen, soil litters content, electrical conductivity, and pH at Qiemo; a strong correlation with soil total nitrogen, soil total nitrogen, and electrical conductivity at Aral; and a strong correlation with soil organic matter, soil total nitrogen, soil water content, and electrical conductivity at Tazhong.

### Environmental factors related to soil GSD

**Figure 5:**
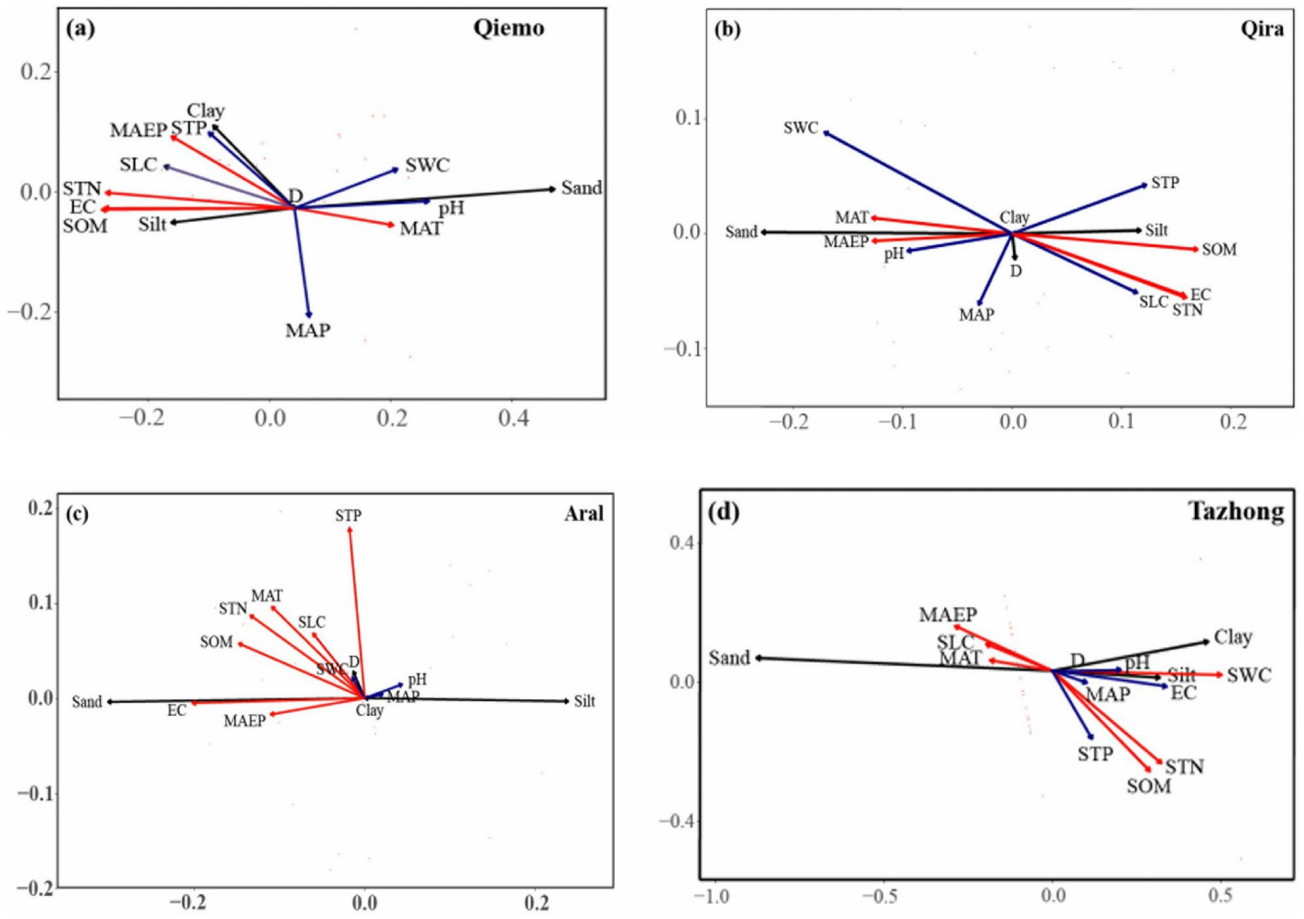
Distance-based redundancy analysis (db-RDA) biplot of soil grain size distribution and environmental factors (MAT, MAP, MAEP, SOM, STN, STP, SLC, SWC, EC, and D represent mean annual temperature, mean annual precipitation, mean annual evaporation, soil organic matter, soil total nitrogen, soil total phosphorus, soil litter content, soil water content, electrical conductivity, and fractal dimension, respectively).

The distance-based redundancy analysis (db-RDA) results are given in Table 3 and Fig. 5. The eigenvalues of soil GSD for the first and second axes were significantly different between sites (Table 3). The eigenvalues of the relationship between soil GSD and environmental factors for the first and second axes were 0.037–0.217 and 0.001–0.048, respectively. At Qiemo, the first axis explained the 91.783% variation of soil GSD and environmental factors (*p* < 0.001), and the second axis explained 8.177% of the variation. At Qira, the first and second axes explained 99.182% (*p <* 0.001) and 0.759% of the variation of soil GSD and environmental factors, respectively. At Aral, the first axis explained 99.417% variation of soil GSD and environmental factors (*p* < 0.05), and the second axis only explained 0.552% of the variation. At Tazhong, the first and second axes explained 98.7571% (*p* < 0.001) and 0.907% of the variation of soil GSD and environmental factors, respectively (Table 3).

**Table 3:** db-RDA of the relationship between soil GSD and environmental factors.

Results	Qiemo		Qira		Aral		Tazhong	
	RDA1	RDA2	RDA1	RDA2	RDA1	RDA2	RDA1	RDA2
Eigenvalues	0.213	0.019	0.037	0.001	0.129	0.001	0.217	0.048
Cumulative proportion of soil grain size	74.975	6.679	49.215	0.376	70.858	0.393	94.436	0.867
Cumulative proportion of soil and environment	91.783	8.177	99.182	0.759	99.417	0.552	98.757	0.907
F-ratio	81.885	7.295	20.515	0.157	51.799	0.287	131.625	3.965
p-value	0.001***	0.733	0.001***	1.000	0.015*	1.000	0.001***	0.981

*p < 0.05, ***p < 0.001.

The fact that the soil grain size arrow pointed approximately the same direction as the environmental factors arrow indicates a strong positive correlation. The arrow lengths of soil organic matter, soil total nitrogen, electrical conductivity, electrical conductivity, mean annual temperature, and mean annual evaporation (the first category of variables) were longer compared than those for the other environmental factors (the second category of variables), suggesting that the first category of variables occupied a larger proportion of the variance of soil GSD. However, soil organic matter, soil total nitrogen, electrical conductivity, mean annual temperature, and mean annual evaporation had a strong influence on soil GSD at all sites (Fig. 5). Soil litter content had a strong positive correlation with the sand content, and a strong negative correlation with the clay and silt contents at Aral and Tazhong (Fig. 5c,d).

## Discussion

Previous studies have indicated that coppice dunes with height > 1 m can capture fine suspension particles (< 50 μm), while saltation affects a wide range of particles (50–500 μm) that are primarily concentrated at the height < 1 m^32,42^. Our data showed that suspension-size particles (< 50 μm) accounted for > 70% of soil GSD on the different *Tamarix* cones (Fig. 2), indicating that suspension plays a dominant role in the *Tamarix* cone formation, even throughout the soil profile. The results of the present study support our hypothesis that suspension-size particles are the main component of the soil GSD. Since the *Tamarix* shrubs can effectively reduce the wind speed, the suspension-size particles are trapped under the shrubs by wind transport, which also explains why silt content is the highest in the formation of *Tamarix* cones^31^. Although the process of saltation cannot be ruled out in the *Tamarix* cone formation, it only makes a very limited contribution to soil PSD on the dunes (Fig. 2). These different contributions of suspension and saltation processes have been observed by both experimental and numerical studies^21,29^. Our studies suggested that the process of creep did not occur on the formation of *Tamarix* cones. In addition, as a result of the size-selective aeolian transport processes, the dune sediments at all sites were poorly sorted except for Tazhong (Fig. 3), presumably related to the deposition of dust sediments of various sizes and the different *Tamarix* shrub sizes at the different sites. The soil GSD exhibited a negative skewness at all sites (Fig. 3), suggesting that the grain size of soil was skewed toward coarse materials.

In this study, silt had the highest particle size fraction (60.60–79.14%), followed by sand (19.14–39.79%) and clay (0–4.81%) (Fig. 2), which is similar to the previous loess studies conducted at the Loess Plateau^10,15,43^. Previous studies indicated that the northeastern part of the Tibetan Plateau, the Badain Jaran Desert, and the Tengger Desert may be the source areas of loess materials of the Loess Plateau^44^. This suggests that the soil particle size of *Tamarix* cones and the typical loesss in the Loess Plateau have the same source, and both may originate from the desert area.

The average height of *Tamarix* cones at all sites was about 3 m in this study, the soil samples collected in the 280–300 cm layer were equivalent to the ground-level soil in the initial stage of the formation of *Tamarix* cones. Previous studies have shown that the mean particle size and content of different particle fractions are all correlated with the dune height^32^. Our data indicate that the soil clay and silt contents decreased with soil depth in the 0–300 cm layer, while the very fine sand and fine sand contents had opposite trends at Qiemo, Qira, and Aral (Fig. S1). This suggests that the suspension-size particles are easily trapped on the top of tall *Tamarix* cones because of long-distance transport by wind, which also explains why the soil fine-grained material (clay and silt) decreases with soil depth. In contrast, soil clay and silt contents at Tazhong increased with soil depth in the 0–300 cm layer (Fig. S1), and the very fine sand content was relatively high. This suggests that soil particle size composition became relatively coarse with the development of *Tamarix* cones, which may be closely related to dust source, wind speed, and the size of *Tamarix* shrubs. In addition, the high content of soil coarse particles in the *Tamarix* cones is likely a combined consequence of sparse vegetation, frequent dust weather, and wind erosion at Tazhong.

Soil fractal dimension is a comprehensive index that can not only mirror the uniformity of soil particle distribution, but also reflect the influence of soil structure and fertility on the soil formation processes^13^. In this study, the fractal dimension was positively correlated with clay and silt contents, and negatively correlated with sand content at all studied soil depths (Table 1), which is consistent with other studies from this region^31,42^. This also support our hypothesis that the fractal dimension can well reflect the particle composition of *Tamarix* cones in the Taklimakan Desert, and the fractal dimension increases with the increase of clay and silt content, and decreases with the increase of sand content (Fig. 4, Table 1). Therefore, the fractal dimension can reflect the degree of soil erosion affected by wind and sand on the *Tamarix* cones, that is, the larger the fractal dimension, the higher the content of fine-grained matter and the weaker the degree of wind erosion, and vice versa.

Among the four study sites, the fractal dimension in the surface soil decreased in the following order: Qiemo > Qira > Aral > Tazhong (Fig. 3). The soil fractal dimension was significantly higher at Qiemo than at the other sites (Fig. 3). Qiemo is located in the transition zone between alluvial plain and desert, which results in fine particles. Moreover, the higher fine particle content may be partly attributed to the different geological formation processes operating in transition zones such as at Qiemo, where the soil is mainly derived from the flood alluvial material, deposited in an earlier geological period and gradually accumulated over time^26^. At Tazhong, the soil fractal dimension was relatively low at 0–100 cm and significantly higher at 100–200 cm (Fig. S1). This indicates that the surface soil was readily affected by wind speed, sand source, and the removal of fine sediment by wind erosion from the surface. These results suggest that the current soil particle size composition is the result of complex physical processes, including the influence of climatic factors, vegetation dynamics, sand source, and micro-topography.

The surface soil composition of the desert environment is the result of the long-term interaction between wind factors and the underlying surface, which mainly depends on the wind velocity and sand source^32^. Therefore, the characteristics of the soil GSD not only reflect the effect of wind on dust transport and sorting in the desert area, but also mirror the influence of obstacles on the transport of wind and sand flow^45^. In this study, the surface soil was dominated by fine particle size at all sites, which was mainly because the large canopies and soft branches of *Tamarix* shrubs were able to intercept fine suspension particles. In addition, the soil particle size composition was mainly composed of fine-grained matter (< 50 μm) in this study (Fig. 2), indicating that the majority of soil particles are in the size range of suspension motion. It can therefore be inferred that dust weather can transport some suspended particles to the study area over long distances.

The soil particle composition is affected by climate, topography, vegetation coverage, and soil properties^15^, Li et al.^46^ studies showed that the soil nutrient content has been shown to increase with the increase of soil fine particles, and soil clay and silt content contribute greatly to soil nutrient content, while soil nutrient content decreases with the increase of coarse sand. In the current study, except for wind velocity, soil GSD was also affected by soil organic matter content, soil total nitrogen content, soil litter content, electrical conductivity, temperature, and evaporation at all sites (Fig. 5), which may be closely correlated with the decomposition of soil litter in the *Tamarix* cones. In the combined action of environmental factors, the decomposition of soil litter can increase the contents of soil fine particles and soil nutrients^26^. Previous studies have shown that the soil fractal dimension can be used to reflect soil fertility, given the significant correlation between the fractal dimension and soil nutrients such as organic carbon, total nitrogen, and total phosphorus^45,47^. In the current study, there was a significant positive correlation with the fractal dimension and soil organic matter, soil total nitrogen, and soil water content throughout the studied soil layers (Table 2). The fractal dimension had a strong correlation with soil organic matter and soil total nitrogen at all sites except Qira (Table S1), which is consistent with the previous studies conducted at regional and national scales^17,32,47,48^. These results show that the fractal dimension can adequately describe the influences of selected soil factors on soil particle compositionin the *Tamarix* cones, and also support our hypothesis that soil GSD and fractal dimension are closely related to soil nutrient, soil water content and soil salinity in the *Tamarix* cones.

The majority of sand dust can be effectively intercepted and fixed by *Tamarix* shrubs in desert areas, thus *Tamarix* cones are formed during long-term dust accumulation processes^22^. Therefore, *Tamarix* cones can serve as a natural quick-sand control system^31^. However, *Tamarix* cones are also an unstable bio-geomorphic landscape, which is prone to decline and degradation in the destruction of vegetation and wind erosion^26^. The Taklimakan Desert, located in the center of Tarim basin, is a major dust source for these areas^48^. These areas have an extremely dry climate with sparse precipitation, intense evaporation, and considerable temperature variation. Annually, the highest wind speed reaches 24.0 m/s and there are more than 200 days with dust weather^36,49^. Therefore, *Tamarix* cones have suffered severe wind erosion and degradation in this area. This is particularly the case in the Tazhong site where, the degradation of *Tamarix* cones is serious due to the sparse vegetation and strong wind erosion on the *Tamarix* cones. In addition, *Tamarix* cones have a tendency to degrade into desert in the oasis–desert ecotone (Qira and Aral sites) due to destructive human activities, which may lead to the aggravation of sand invasion and the degradation of regional environment. Therefore, the protection of *Tamarix* cones should be strengthened to achieve a stable ecological environment in the desert areas.

## Conclusions

In this study, the vertical distributions of soil GSD and their parameters varied among sites as a result of the complex interactions among vegetation, micro-climate, and soil proprieties, and the formation process of *Tamarix* cones. In the *Tamarix* cones of the Taklimakan Desert, soil particle composition was dominated by silt and very fine sand. Suspension-size particles are the main component of the soil GSD and decrease with the increasing depth in the profiles at the *Tamarix* cones, and that the fractal dimension can reflect the characteristics of the soil GSD. The strong relationships between the fractal dimensions and selected soil properties indicate that the fractal dimension can quantify changes in soil properties. It also can suggest degree of desertification and degradation in the desert region. In addition, As vegetation coverage decreased, the fractal dimension correspondingly decreased because of the increase of coarse particles. Furthermore, the formation process of *Tamarix* cones also affected the soil GSD to some extent. Our data provide supplementary information relating to the soil formation processes of coppice dunes for use in conservation and management of *Tamarix* cones in extreme arid desert ecosystems.

## Supplementary Information

Supplementary Information 1.

Supplementary Information 2.

## Data Availability

The data used to support the findings of this study are included within the article. We declare that experimental research and field studies on plants comply with relevant institutional, national, and international guidelines and legislation. In addition, we confirm that all methods were carried out in accordance with relevant guidelines in the “Materials and methods” section.
