# Supplementary material for: Fractal features of soil grain-size distribution in a typical Tamarix cones in the Taklimakan Desert, China
Source: Sci Rep. 2022 Sep 30;12:16461. doi: 10.1038/s41598-022-20755-x (PMC9525648; doi:10.1038/s41598-022-20755-x)
Supplement: Supplementary file 1 — Supplementary Information 1. [file 41598_2022_20755_MOESM1_ESM.doc]

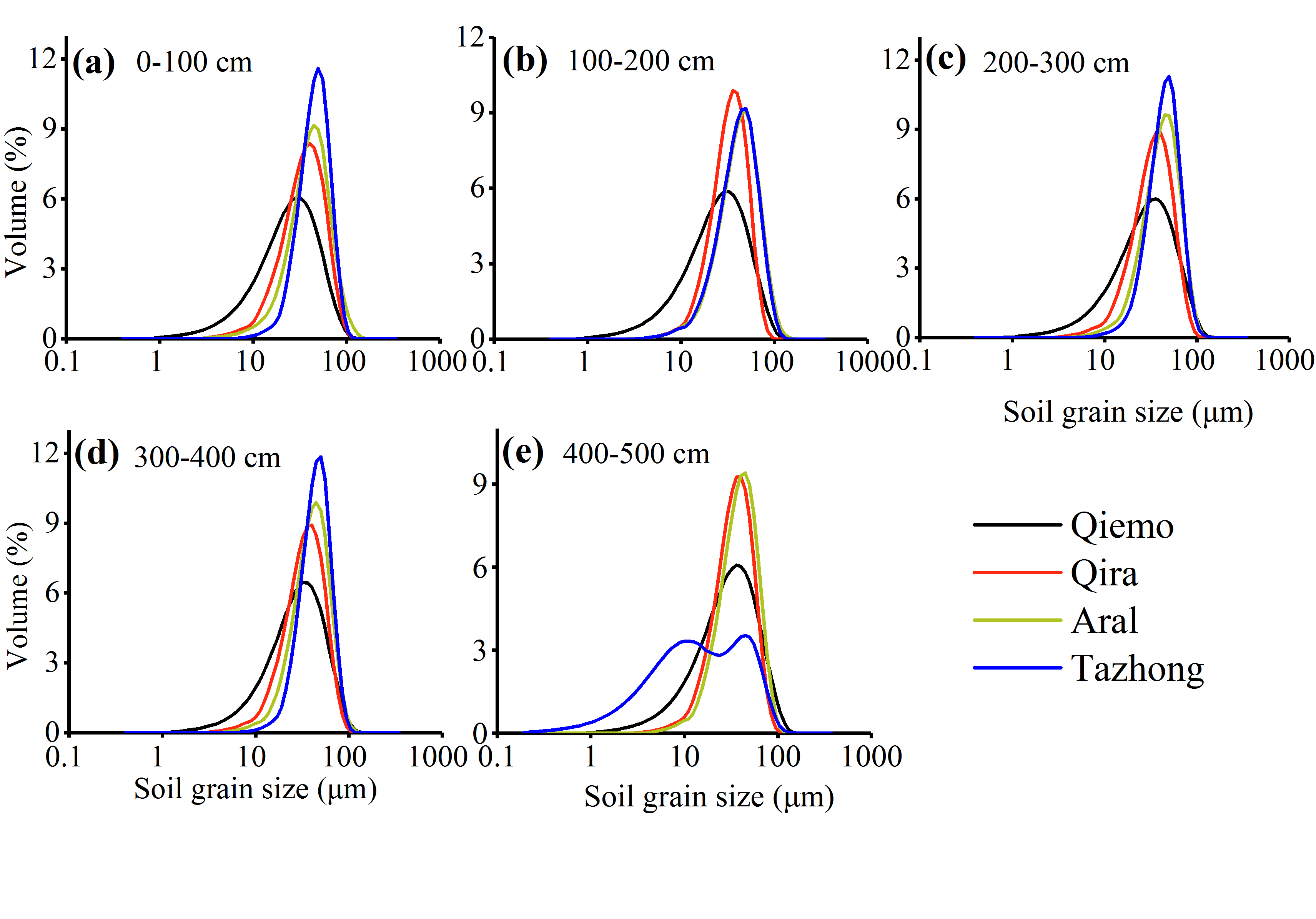
**Figure S1.** Characteristics of grain size distribution in the different soil layers.

**Table S1.** Correlations among the fractal dimension, clay, silt, and sand contents and environmental factors in the different habitats.

| Sites | Grain size | MAT | MAP | MAEP | SOM | STN | STP | SLC | SWC | pH | EC |
| --- | --- | --- | --- | --- | --- | --- | --- | --- | --- | --- | --- |
| Qiemo | Clay | -0.342 | -0.389 | 0.612** | 0.606** | 0.623*** | 0.443* | 0.452* | -0.122 | -0.441* | 0.572** |
|  | Silt | -0.440* | -0.007 | 0.525** | 0.870*** | 0.855*** | 0.344 | 0.526** | 0.456* | -0.607** | 0.856*** |
|  | Sand | 0.453* | 0.035 | -0.555** | -0.891*** | -0.877*** | -0.368 | -0.545** | -0.452* | 0.622*** | -0.874*** |
|  | Fractal dimension | -0.315 | -0.210 | 0.641*** | 0.647*** | 0.653*** | 0.383 | 0.466* | -0.193 | -0.522** | 0.613** |
| Qira | Clay | — | — | — | — | — | — | — | — | — | — |
|  | Silt | -0.421* | -0.115 | -0.411* | 0.559** | 0.511** | 0.398* | 0.349 | 0.572** | -0.290 | 0.531** |
|  | Sand | 0.421* | 0.115 | 0.411* | -0.559** | -0.511** | -0.398* | -0.349 | -0.572** | 0.290 | -0.531** |
|  | Fractal dimension | -0.095 | 0.158 | -0.034 | 0.117 | 0.230 | -0.078 | 0.226 | -0.313 | 0.031 | 0.238 |
| Aral | Clay | — | — | — | — | — | — | — | — | — | — |
|  | Silt | -0.371 | 0.036 | -0.387 | -0.497* | -0.452* | -0.066 | -0.220 | -0.077 | 0.152 | -0.681*** |
|  | Sand | 0.371 | -0.036 | 0.387 | 0.497* | 0.452* | 0.066 | 0.220 | 0.077 | -0.152 | 0.681*** |
|  | Fractal dimension | 0.495* | -0.037 | 0.127 | 0.461* | 0.514** | 0.632*** | 0.367 | 0.116 | -0.024 | 0.319 |
| Tazhong | Clay | -0.303 | 0.137 | -0.492* | 0.475* | 0.477* | 0.126 | -0.286 | 0.913*** | 0.358 | 0.617** |
|  | Silt | -0.384 | 0.155 | -0.608** | 0.629*** | 0.668*** | 0.306 | -0.389 | 0.933*** | 0.404* | 0.730*** |
|  | Sand | 0.373 | -0.153 | 0.592** | -0.660*** | -0.637*** | -0.273 | 0.373 | -0.940*** | -0.400* | -0.716*** |
|  | Fractal dimension | -0.365 | 0.136 | -0.242 | 0.753*** | 0.769*** | 0.480* | -0.348 | 0.671*** | 0.203 | 0.798*** |

* *p* < 0.05, ** *p* < 0.01, *** *p* < 0.001. SOM = soil organic matter, STN = soil total nitrogen, STP = soil total phosphorus, SLC = soil litter content, SWC = soil water content, EC = electrical conductivity.
